# Optimization and Effect of Water Hardness for the Production of Slightly Acidic Electrolyzed Water on Sanitization Efficacy

**DOI:** 10.3389/fmicb.2022.816671

**Published:** 2022-03-02

**Authors:** Pianpian Yan, Hyeon-yeong Jo, Ramachandran Chelliah, Kyoung hee Jo, Nam Chan Woo, Min Seung Wook, Deog Hwan Oh

**Affiliations:** ^1^Department of Food Science and Biotechnology, College of Agriculture and Life Sciences, Kangwon National University, Chuncheon-si, South Korea; ^2^Seoulin Bioscience Company, Seongnam-si, South Korea

**Keywords:** geographical location, water hardness, sanitization efficacy, pH, storage condition

## Abstract

Slightly acidic electrolyzed water (SAEW) has been recently proposed as a novel promising sanitizer and cleaner in the agricultural and food industries. However, several factors, including water hardness, were considered to strongly affect the physical properties and sanitization efficacy of SAEW. To study the effect of water hardness on the SAEW production, we evaluated the production properties and sanitization effect of SAEW, which was generated from water sources in 16 representatively geographical locations of South Korea. The results showed that the hardness of water sources from Kangwon-do, Jeollanam-do, and Daegu was 22–41 ppm; that from Busan, Gyeongnam-do, Gwangju Bukgu was 80–443 ppm, and that from seven other locations was 41–79 ppm. SAEW is produced from water hardness less than 50 ppm and greater than 80 ppm was beyond the accepted pH range (5.0–6.5). Notably, high-hardness water (>80 ppm) containing 5% HCl could be used to produce SAEW with accepted pH. The SAEW generated from low-hardness water with additions of 2% HCl and 2 M NaCl at 7 A showed accepted pH and higher germicidal effect. Furthermore, SAEW with the available chlorine concentration of 27–41 mg/L for 1 min was sufficient to completely inactivate non–spore-forming foodborne pathogens. Sanitization efficacy was not markedly affected by storage conditions for SAEW at 40 ppm. Our results demonstrated that the degree of water hardness is an important factor in the production of SAEW, which would provide a foundation for commercial application of SAEW.

## Introduction

Foodborne disease refers to any illness caused by contaminated food spoiled by pathogenic bacteria, parasites, and viruses (Adley and Ryan, 2016; Fung et al., 2018). Foodborne diseases, causing health concerns and economic losses, are still difficult to control worldwide (Grace et al., 2020). Sanitation techniques provide good opportunities for defense against foodborne pathogens in the agriculture and food industries. Numerous commercial sanitizers, such as physical technology (ozone), chemical technology (chlorine compounds and peroxide mixtures), and biological technology (essential oil), have been used as disinfection agents throughout the food supply chain (Aslam et al., 2021; da Costa Lima and de Souza, 2021; Jones et al., 2021). However, some of these technologies in the food industries are not entirely acceptable because of their disadvantages, including chemical residues, limited inactivation, adverse effects on food quality, and potential toxicity to human beings or the environment (Marshall et al., 2020). Thus, it is urgent to develop safe and effective disinfectants in the food industries.

Slightly acidic electrolyzed water (SAEW) has been proposed as a novel promising sanitizer and cleaner in recent years (Rahman et al., 2016). SAEW is a solution produced by electrolyzing dilute electrolyte (usually contain NaCl and/or HCl) in an electrolysis chamber without diaphragm, which contains the anode and cathode electrodes shown in the Supplementary Figures 1, 2 (Xuan and Ling, 2019). SAEW with neutral pH (5.0–6.5) exhibits high antimicrobial activity because it contains a large amount of hypochlorous acid. Meanwhile, it has the advantages of reducing the corrosion to food-processing plants and less damage to the environment (Aider et al., 2012; Yan et al., 2021; Zhao et al., 2021). Furthermore, electrolyzed water (EW) showed antimicrobial activity against a wide range of microorganisms and eliminated the most common form of bacteria, fungi, viruses, and spores in food, food-processing surface, and non–food-contact surfaces in a relatively short time (usually within 10–20 s) (Phuvasate and Su, 2010; Zheng et al., 2013; Possas et al., 2021).

Commercial SAEW is extensively used in food manufacture because of its advantages. However, some studies have demonstrated that the properties of SAEW are affected by factors such as type and concentration of electrolyte, current, flow rate, water source, and storage condition (Kim et al., 2019). To date, studies focused only on the sanitizing effect and production of SAEW using one type of starting water in standardized settings; the property (pH) of water was not investigated. The water hardness may be an important factor to influence the production of SAEW in the starting water because of its strict pH range (5.0–6.5) (Zhang et al., 2021). In addition, storage of SAEW can also influence the physicochemical properties and bactericidal activity of SAEW (Xuan et al., 2016). However, the storage condition of SAEW on the bactericidal activity and the properties of SAEW were not fully studied.

The objective of this study are (1) To study the effects on the production of SAEW including pH and ACC (available chlorine concentration) using tap water collected in different geographical locations in South Korea; (2) To optimize production conditions of SAEW from low- and high-hardness-water source and to measure the efficacy of SAEW in activating foodborne pathogens; (3) To compare stability (pH and ACC) and bactericidal efficiency of SAEW (20 and 40 ppm) under different storage conditions (open and close) after 6 months. Our results aid to understanding the generation system, physicochemical properties, and bactericidal activity of SAEW, which would contribute to the enhancement of the practical application of SAEW in the agriculture and food industries.

## Materials and Methods

### Collection of Water in the South Korea

A total of 16 water samples were collected from 16 sites in South Korea, shown in the Supplementary Figure 3. The collection of tap water was preferentially sampled from representative drinking water reservoirs and populous cities and from a large number of local municipalities. The collection place was Seoul, Incheon, Daejeon, Busan, Daegu, Gwangju, Gyeonggi-do, Chungbuk-do (Cheonju), Gangwon-do (chuncheon), Jeonnam-do (Gwangju, Gwangyang, and Gwangju Bukgu), Jeonbuk-do (Jeongfeup), Gyeongnam-do (Jinju and Haman). The volume of tap water of each sample is 40 L. The samples were stored at room temperature until analysis.

### Bacterial and Preparation of Inoculum

*Escherichia coli* O157:H7 (ATCC 43895), *Bacillus cereus* (ATCC 10987), *Listeria monocytogenes* Scott A (ATCC 43251), *Staphylococcus aureus* (ATCC 13565), *Salmonella* enteritidis (ATCC 13076), and *Clostridium perfringens* (ATCC 13124) were used in this study. Stock cultures were resuscitated by culturing in brain heart infusion broth (Becton Dickinson Diagnostic Systems, Sparks, MD, United States) for 24 h at 37°C. The working bacterial concentration was approximately 8 log colony-forming units (CFU)/mL.

### Slightly Acidic Electrolyzed Water Primary Preparation

The SAEW generator used in this study was supplied by Seoulin Bioscience (ecoTree^®^, Seongnam, South Korea). The SAEW-producing machine was provided by Seoulin Bioscience Company (South Korea). The initial SAEW was produced by electrolysis of 3% diluted hydrochloric acid solution in a chamber cell without membrane at a setting current of 6 A. The electrolytic cell (80 m × 12.5 mm × 0.5 T) contained both cathode (Ti) and anode (IrO_2_). Water samples from different places were injected to the number 4 bottles at the flow rate of 1.5 mL/min, shown in Supplementary Figure 2. The SAEW was collected after the amperage of generation has stabilized for 15 min.

### Optimization of Slightly Acidic Electrolyzed Water Production Conditions

Collection of water from Kangwon National University region (low water hardness = 25 ppm) and Gwangju Bukgu region (high water hardness = 212 ppm) was used to optimize SAEW production. The water sample flow rate remained at 4 L/min, whereas 2, 3, and 5% HCl were combined with each of 0, 1, and 2 M NaCl at 6, 7, and 8 current and was injected into the number 4 bottle at the flow rate of 1.5 mL/min (low-hardness-water source). The concentration of 2, 3, and 5% HCl is optimized for high-hardness-water source. The antimicrobial efficacy of SAEWs was evaluated *in vitro* using *C. perfringens* (ATCC 13124), *E. coli* O157:H7 (ATCC 43895), *B. cereus* (ATCC 10987), *L. monocytogenes* Scott A (ATCC 43251), *S. aureus* (ATCC 13565), and *Salmonella* enteritidis (ATCC 13076) broth cultures.

### Determination of pH, Available Chlorine Concentration, and Water Hardness

Water hardness was analyzed by Korean Capital Research Institute according to the manufacturer’s instructions. The pH values of all produced SAEWs were determined using a dual-scale pH meter (Accumet model 15; Fisher Scientific Co., Hampton, VA, United States). The ACC of all SAEW was measured by a colorimetric method with a digital chlorine test reagent (20J3A; Kasahara Chemical Instruments Corp., Japan). The detection range was from 0 to 300 mg/L.

### *In vitro* Sanitization Treatments

The selected pathogens were inactivated *in vitro* using the method according to Forghani et al. (2015). One milliliter of each selected bacterial cell suspension was added to 9 mL SAEW, and the tube was shaken immediately for 1-min contact time. One milliliter of each sample was transferred to a 9 mL of neutralizing solution tube (0.5% sodium thiosulfate ±0.85% sodium chloride) and reaction of 1 min to stop SAEW decontamination activity after treatment. Samples were serial dilutions (1:10) in 9 mL of buffered peptone water (0.1% BPW; Difco, Sparks, MD, United States).

### *In vivo* Sanitization Treatments

Fresh spinach, chicken, and pork samples were weighed 10 g to be transferred into a sterile plastic bag and used it within 1 h. Contamination inoculum was prepared using 0.1% peptone water adjusting the bacterial count to 10^6^ CFU/mL. Spinach, chicken, and pork were inoculated with *E. coli* O157:H7, *Salmonella* enteritidis, and *L. monocytogenes* Scott A, respectively, for 15 min. Then, the inoculated spinach, chicken, and pork were, respectively, immersed in 90 mL SAEW in the sterile containers (Whirl-Pak, United States). After 5-min SAEW treatment, the reaction was stopped by addition of 200 mL neutralizing solution.

### Viability Measurements

The untreated bacterial cell (*S. aureus* and *E. coli*) and treated samples (SAEW) were centrifuged (4,000*g* for 10 min at 4°C) and suspended in 0.1% BPW. The dead and live bacterial cells were stained with propidium iodide (PI) and Syto-9 (SYT), respectively (LIVE/DEAD BacLight™ Bacterial Viability Kit; Molecular Probes, Invitrogen). The excitation and emission wavelength of PI and SYT was 493–636 and 504–523 nm, respectively. The staining was completed in the dark condition at room temperature for 30 min. Fluorescence microscopic imaging of the cells was performed using a fluorescence Olympus IX51 microscope (U-LH100HG; Olympus Corporation, Tokyo, Japan).

### Procedure of Storage Experiment

Two different concentrations were designed for experiment, including 41 and 25 ppm. Two types of HDPE bottles (closed and open) were used to collect the SAEW samples described above. The four samples were stored at a room temperature of 25°C for 6 months. It was used for the experiments for exposure to light. The pH and ACC of the samples were measured on storage months 0, 1, 2, 3, 4, 5, and 6. The bacterial experiment was measured on storage months 0 and 6 by using *E. coli* O157:H7 (ATCC 43895), *B. cereus* (ATCC 10987), *L. monocytogenes* Scott A (ATCC 43251), *S. aureus* (ATCC 13565), and *Salmonella* enteritidis (ATCC 13076) broth culture. The measurements were completed within 30 min. Each experimental was repeated in triplicate.

### Statistical Analyses

Statistical analysis (mean values of microbial populations, ACC, pH from each treatment, and measurement) was performed using sing IBM SPSS Statistics version 19 (SPSS Inc., An IBM Company, Chicago, IL, United States). The significance of difference was defined at *p* ≤ 0.05 using Tukey multiple-range tests.

## Results

### Distribution of Tap Water Hardness in the South Korea

The water samples, used to produce SAEW, were collected from drinking water from four major rivers [Paldang (Han River), Mulguem (Nakdong River), Daechong (Geum River), and Juam (Yeongsan River)] in South Korea in 2019 from 16 places. Sixteen representatively geographical locations were selected to collect water, and the hardness of water samples was tested and summarized in Supplementary Figure 3. The result showed that the hardness of most water samples was 54–80 ppm, and water samples from Gangwon-do and Jeollanam-do were 22–26 ppm in the hardness. On the contrary, the hardness of water samples from Gwangju Bukgu and Haman area is 212 and 443 ppm, respectively, because of using groundwater rather than tap water.

### Effect of Variety of Water on Slightly Acidic Electrolyzed Water Properties

**FIGURE 1:**
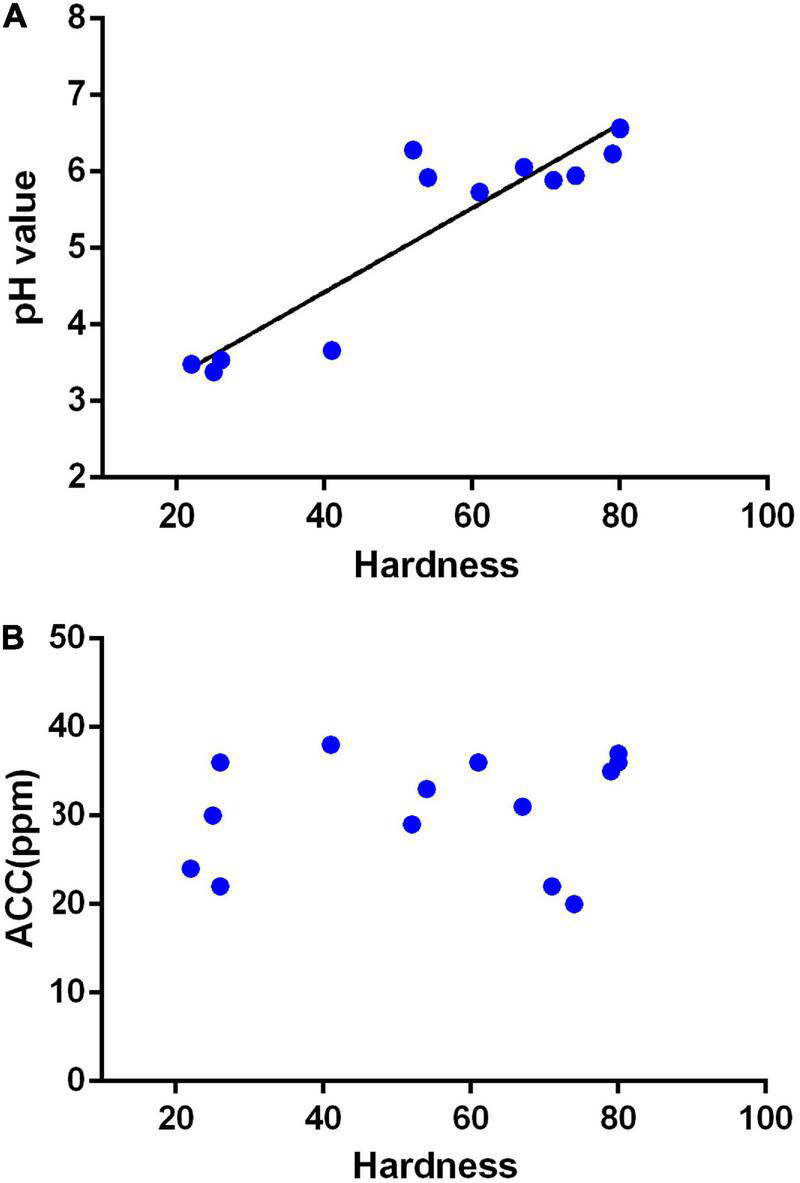
Relationship between the hardness of tap water to the available chlorine concentration (ACC) and pH of slightly acidic electrolyzed water (SAEW). **(A)** Relation of tap water hardness to pH; *Y* = 0.05492*x* + 2.227, *R* = 0.8655. **(B)** Relation of tap water hardness to ACC. The data were analyzed by the tap water hardness except for underground water.

The characteristics of SAEW produced in different places with the pH range of 6.61–7.83 and the water hardness range of 22–443 ppm are shown in Table 1. All generator settings and other conditions were also the same for above waters. It is well-known that the pH range of SAEW is 5–6.5, and the available chlorine concentration is 10–80 mg/L. The ACCs of all collected samples are within the acceptable range from 20 to 38 ppm. Interestingly, the EW was produced from Gangwon-do, Daegu, Gwangju, and Gwangyang (hardness <50 mg/L) could not reach a pH of 5.0, whereas the EW from Gwangju Bukgu, Busan Seogu, Jinju, and Haman (hardness > 80 mg/L) was higher than pH 6.5. However, the acceptable SAEW can be produced from those places with hardness range of greater than 52–79 mg/L. Furthermore, we also investigated the relationship between ACC and pH of EW, generated from water samples with different hardness (Figure 1). It was shown that the pH of electrolytic water is significantly related to the hardness of tap water. This result showed that hardness was an important factor affecting the properties of EW, and the system and electrolyte need to be further optimized.

**TABLE 1:** Properties of produced EWs based on the collected water samples from South Korea.

Sample no.	Properties of tap water			Electrolyzed water	
	Place	pH	Water hardness	pH	ACC
1	Incheon	7.03	67	6.06	31
2	Seoul	6.84	71	5.89	22
3	Gangwon-do (chuncheon-1)	6.94	25	3.38	30
4	Gangwon-do (chuncheon-2)	7.18	26	3.54	36
5	Gyeonggido-Hwaseong-1	7.72	74	5.95	20
6	Gyeonggido-Hwaseong-2	7.30	52	6.28	29
7	Chungbuk (Cheonju)	6.81	54	5.92	33
8	Daejeon	7.30	79	6.23	35
9	Jeonbuk (Jeongeup)	7.28	61	5.73	36
10	Daegu	7.08	41	3.66	38
11	Gwangju	6.61	26	3.56	22
12	Jeonnam-do (Gwangyang)	6.69	22	3.48	24
13	Gyeongnam-do (Jinju)	7.83	80	6.56	36
14	Gyeongnam-do (Haman)	7.51	443	6.74	27
15	Busan	7.01	80	6.57	37
16	Jeonnam-do (Gwangju Bukgu)	7.32	212	6.78	34

*Current: 6A; flow rate: 1.5 L/min; electrolyte: 3% HCl.*

### Optimization of Condition to Produce Slightly Acidic Electrolyzed Water From Tap Water With Low Hardness

**FIGURE 2:**
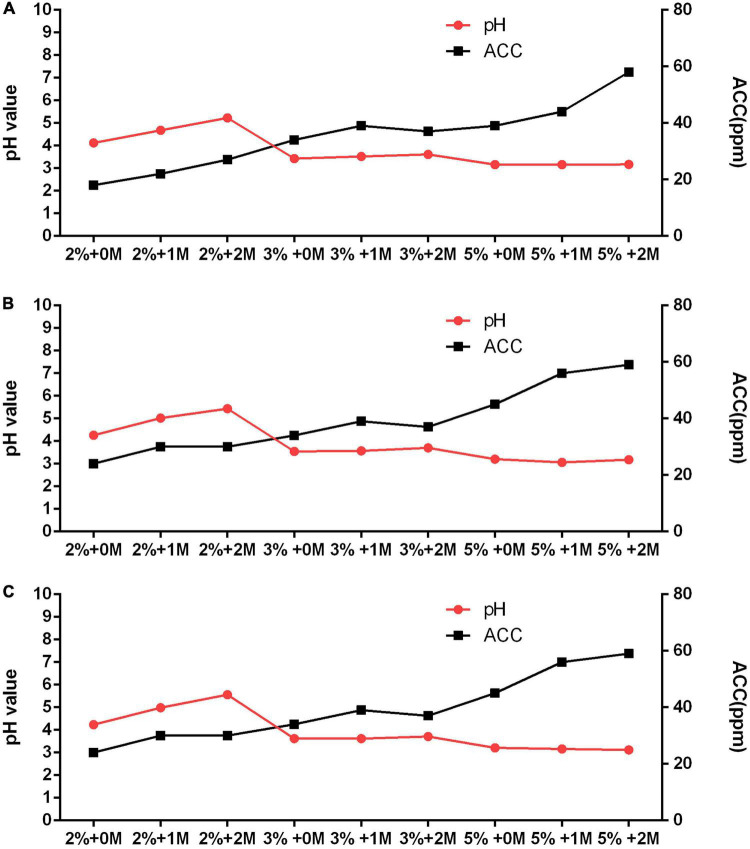
Optimization of slightly acidic electrolyzed water (SAEW) production system with different electrolyte concentrations and current using low-hardness water 25 ppm. **(A)** 6 current. **(B)** 7 current. **(C)** 8 current.

The water sample (hardness = 25 mg/L) in Chuncheon was selected to further explore optimal current, electrolyte concentration, and salt to produce acceptable SAEW. A variety of EWs (SAEW, acidic) were generated from combination of each 2, 3, and 5% HCl, and 0, 1, and 2 M NaCl, together with 6, 7, and 8 current of the production system, which is shown in Figure 2 and Table 2. The results showed that the current did not significantly affect the preparation of EW. Regardless of the type of produced EW, increasing the concentration of electrolyte resulted in higher concentration of ACC in the generated EW. The ACC and pH values of the produced EWs ranged from 18 to 59 ppm and 3.06 to 5.56, respectively. Only three among the total 27 different types of generated EWs could be regarded as SAEW. What is more, with an addition of 2 M NaCl together with 2% HCl, the 6, 7, and 8 current could be used to produce SAEWs that showed pH 5.23, 5.44, and 5.56 and ACC 27, 30, and 30, respectively. The addition of 2 M NaCl + 2% HCl at 6 and 7 A showed higher AAC (30 ppm). Furthermore, application of the SAEW (2 M NaCl + 2% HCl + 7A) resulted in 8.37 ± 0.02, 8.38 ± 0.04, 8.36 ± 0.06, 8.27 ± 0.03, 8.23 ± 0.01, and 3.93 ± 0.34 (log CFU/mL) reduction in the numbers of *E. coli*, *Salmonella* enteritidis, *L. monocytogenes*, *S. aureus*, *B. cereus*, *C. perfringens*, respectively. Therefore, the electrolyte of 2 M NaCl + 2% HCl at 7A was the optimized condition to produce the SAEW from tap water with low hardness.

**TABLE 2:** Antimicrobial effect of different electrolytic and current using low-water-hardness water source.

Current (A)	Electrolyte		pH	ACC	Log reduction (log_10_ CFU/mL)					
	HCl (%)	NaCl (mol/L)			EC	SE	LM	SA	BC	CLP
6.0	2	0	4.12	18	ND	ND	ND	ND	4.86 ± 0.13^a^	4.68 ± 0.01^ab^
		1	4.68	22	ND	ND	ND	ND	4.78 ± 0.11^a^	4.36 ± 0.26^bc^
		2	5.23	27	ND	ND	ND	ND	ND	3.93 ± 0.03*^def^*
	3	0	3.43	34	ND	ND	ND	ND	ND	3.31 ± 0.08^ghi^
		1	3.52	39	ND	ND	ND	ND	ND	3.26 ± 0.05^hi^
		2	3.61	39	ND	ND	ND	ND	ND	3.27 ± 0.03^hi^
	5	0	3.16	39	ND	ND	ND	ND	ND	3.24 ± 0.04^i^
		1	3.16	44	ND	ND	ND	ND	ND	2.76 ± 0.08^j^
		2	3.17	58	ND	ND	ND	ND	ND	ND

7.0	2	0	4.26	24	ND	ND	ND	ND	ND	4.31 ± 0.01^bcd^
		1	5.02	30	ND	ND	ND	ND	ND	3.90 ± 0.02*^de^*
		2	5.44	30	ND	ND	ND	ND	ND	3.93 ± 0.34*^def^*
	3	0	3.55	34	ND	ND	ND	ND	ND	3.92 ± 0.03^ef^
		1	3.57	39	ND	ND	ND	ND	ND	3.23 ± 0.08^i^
		2	3.70	39	ND	ND	ND	ND	ND	3.30 ± 0.03^ghi^
	5	0	3.20	45	ND	ND	ND	ND	ND	2.74 ± 0.07^j^
		1	3.26	56	ND	ND	ND	ND	ND	ND
		2	3.28	59	ND	ND	ND	ND	ND	ND

8.0	2	0	4.24	26	ND	ND	ND	ND	ND	4.16 ± 0.01^cde^
		1	4.99	30	ND	ND	ND	ND	ND	3.86 ± 0.07^ef^
		2	5.56	30	ND	ND	ND	ND	ND	3.88 ± 0.05^ef^
	3	0	3.62	36	ND	ND	ND	ND	ND	3.30 ± 0.01^ghi^
		1	3.62	39	ND	ND	ND	ND	ND	3.89 ± 0.07^ef^
		2	3.71	40	ND	ND	ND	ND	ND	3.67 ± 0.03^fg^
	5	0	3.12	51	ND	ND	ND	ND	ND	3.64 ± 0.02^fgh^
		1	3.16	68	ND	ND	ND	ND	ND	ND
		2	3.16	71	ND	ND	ND	ND	ND	ND

*Tap water: Kangwondo (Chunchon) pH 6.94, hardness 25 ppm.*

*EC, SE, LM, SA, BC, CLP: 8.37 ± 0.02, 8.38 ± 0.04, 8.36 ± 0.06, 8.27 ± 0.03, 8.23 ± 0.01, 8.67 ± 0.10 log CFU/mL.*

*Current (ampere); ACC (ppm). Voltage: 2.4 V; flow rate: 1.7 L/min. Bars labeled with different letters in the same reduction group show a significant difference (p < 0.05).*

*EC, E. coli; SE, Salmonella enteritidis; LM, L. monocytogenes; SA, S. aureus; BC, B. cereus; CLP, C. perfringens; ND, not detected.*

### Optimization of Condition to Produce Slightly Acidic Electrolyzed Water From Tap Water With High Hardness

The tap water (hardness = 212 ppm) in Gwangju Bukgu was selected to further explore optimal hypochlorous acid concentration to produce acceptable SAEW. The ACCs and pH values of the produced EWs ranged from 34 to 46 and 5.87 to 6.78, respectively. The pH is acceptable to produce SAEW after addition of 4 and 5% HCl, which ACC from EWs was 42 and 46 ppm, respectively, is shown in the Supplementary Figure 4.

### Effect of Slightly Acidic Electrolyzed Water Sanitization Efficacy

**FIGURE 3:**
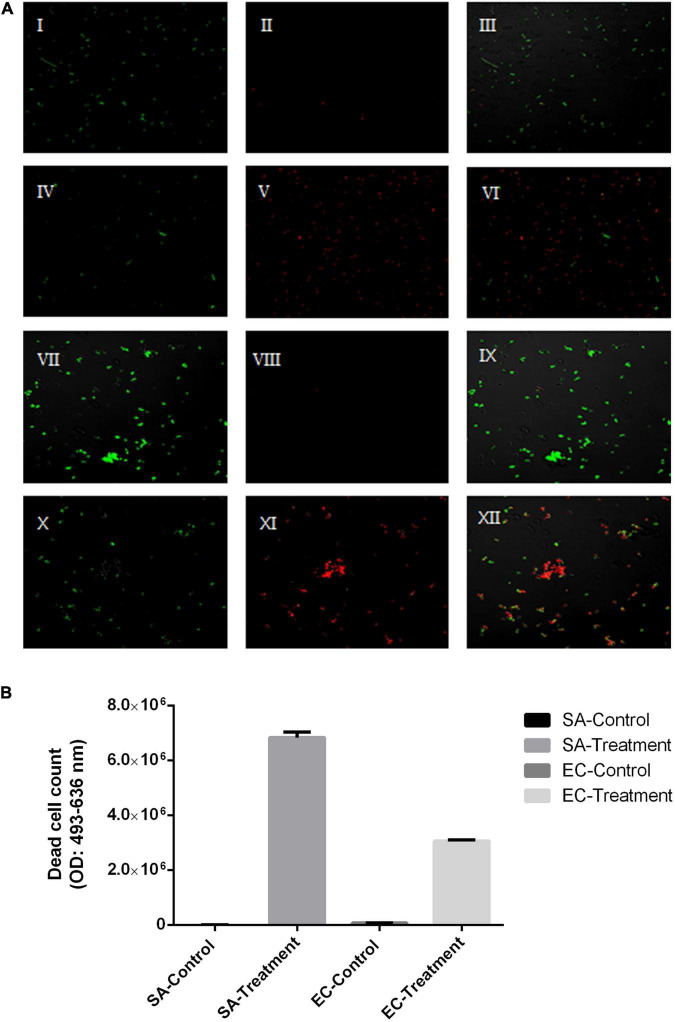
**(A)** Live/dead fluorescent staining of *Escherichia coli* and *Staphylococcus aureus* control samples and after a 1-min slightly acidic electrolyzed water (SAEW) at 40 ppm. **(B)** The count of dead cells as assayed by DEAD cell staining. Green-stained are live cells, and red-stained are dead cells. Live cells: single syto9 staining (I, IV, XII, X), dead cells: single PI staining (II, V, VIII, XII), live and dead cells: Syto9 + PI staining (III, VI, IX, XIII).

To evaluate its sanitization efficacy, the antimicrobial effect of SAEW produced from water samples with middle hardness was further studied. The result showed that the reduction in the numbers of *C. perfringens*, *E. coli*, *Salmonella* enteritidis, *L. monocytogenes*, *S. aureus*, *B. cereus* was 3.62 ± 0.02, 8.27 ± 0.03, 8.22 ± 0.09, 8.47 ± 0.10, 8.35 ± 0.07, and 8.23 ± 0.60 (log CFU/mL), respectively (Table 3). In addition, Figure 3 shows the live/dead cells fluorescence staining images of tested pathogens after 1-min SAEW treatment. *E. coli* and *S. aureus* were both sensitive to a 1-min SAEW treatment. Most of the cells in the control samples appeared fluorescent green, from which they could be redistributed as separated spots. However, red blood cells were observed in the SAEW samples.

**TABLE 3:** Antimicrobial effect of different electrolytic and current using middle-water-hardness water source.

Electrolyte		Current (A)	pH	ACC	Log reduction (log_10_ CFU/mL)					
HCl (%)	NaCl (mol/L)				EC	SE	LM	SA	BC	CLP
3	0	6	6.01	41	ND	ND	ND	ND	ND	3.62 ± 0.02^a^

*Bars labeled with different letters in the same reduction group show a significant difference (p < 0.05).*

*Tap water hardness: 40 ppm.*

*EC, E. coli; SE, Salmonella enteritidis; LM, L. monocytogenes; SA, S. aureus; BC, B. cereus; CLP, C. perfringens;*

*ND, not detected.*

**FIGURE 4:**
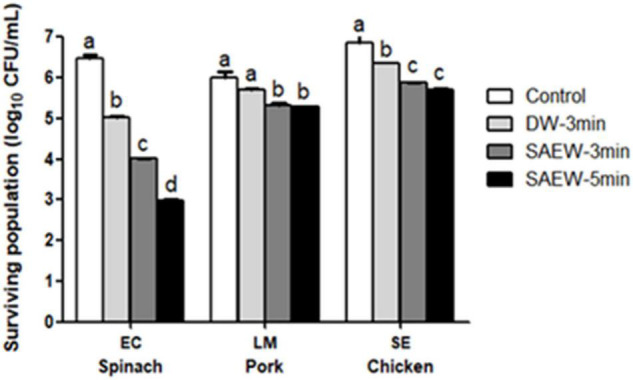
The impacts of slightly acidic electrolyzed water (SAEW) on inactivation inoculated pathogens artificially on food samples. Control, untreated samples; LM, *L. monocytogenes*; SE, *Salmonella* enteritidis; EC, *E. coli*. Pathogen inactivation contact time: 3 and 5 min. Bars labeled with different letters in the same reduction group show a significant difference (*p* < 0.05).

Selection of following three different pathogens was performed according to natural distribution on the samples. *E. coli* O157:H7 is a common pathogen of raw vegetables, as well as many other types of food (Mukhopadhyay et al., 2019). *Salmonella* enteritidis occurs frequently in raw chicken pork meat, whereas *L. monocytogenes* is concerning pathogens in meats (Heredia and García, 2018). Figure 4 shows the inactivation of pathogens artificially inoculated on food samples using SAEW treatment with different dipping time (3 and 5 min). Both SAEW treatments could significantly reduce the microbial numbers compared with water treatment (*p* < 0.05). The counts of *E. coli*, *L. monocytogenes*, and *Salmonella* enteritidis in spinach, pork, and chicken decreased by 3.4, 0.71, and 1.45 log CFU/g, respectively, after the 3-min sanitization treatment. In comparison, the 5-min sanitization treatment is significantly higher on spinach from 3-min treatments. There was no significant sanitization efficacy difference on beef and chicken between 3 and 5 min. The bactericidal efficacy of EW is limited by the following factors: the presence of organics materials (lipids, proteins, and so on) (Jo et al., 2018). Therefore, this SAEW should be considered effective bactericidal treatment strategies to ensure the safety of food samples.

### Properties of Slightly Acidic Electrolyzed Water During Storage

**FIGURE 5:**
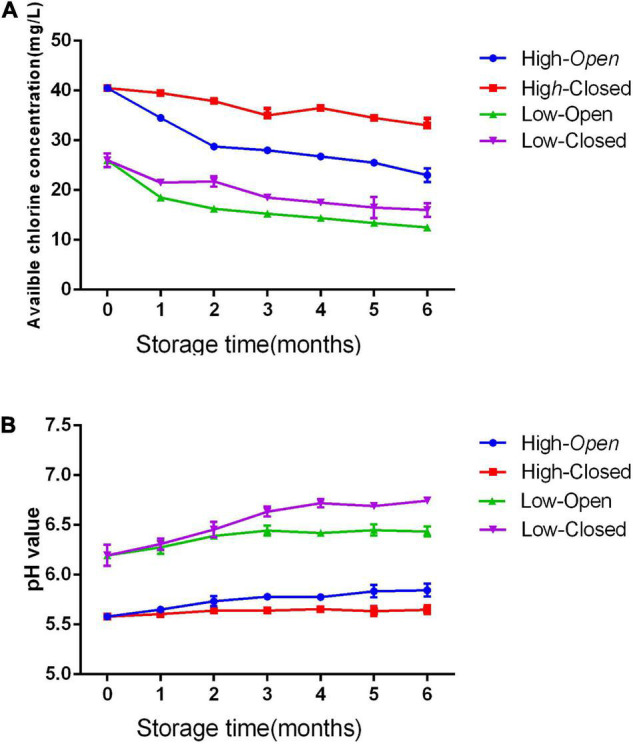
Effect of storage condition on pH **(A)** and available chlorine concentration (ACC) **(B)** value of slightly acidic electrolyzed water (SAEW).

The changes in pH and ACC of SAEW at two concentrations (20 and 41 ppm) under two different storage conditions (open and closed) are shown in Figure 5. The EWs (20 and 41 ppm) stored under the closed condition were more stable with a minimum reduction in ACC, whereas a greater loss of chlorine was found under the open condition (difference of -61.42% for SAEW). In addition, the SAEWs (20 and 41 ppm) under open condition slightly increased pH value after a 6-month storage. However, the pH of SAEW remained stable in the range of 5.60–5.68 and 6.27–6.42 in the two different concentrations under the closed condition, respectively. The concentration had no significant effect on the physicochemical properties of SAEW (*p* > 0.05). This result suggests that the storage of SAEW in a closed container contributes to prevent the loss of chlorine, which is one of the main factors of antimicrobial activity.

### Bactericidal Efficiency of Slightly Acidic Electrolyzed Water Before and After Storage

The bactericidal efficiency of low and high concentrations of SAEW (before and after the 6-month storage) to inactivate *E. coli*, *Salmonella* enteritidis, *L. monocytogenes*, *S. aureus*, and *B. cereus* was evaluated. Following the 6-month open and closed storage, the SAEW at 20 ppm showed 4.57 and 4.44 (log10 CFU/mL) reduction of *B. cereus*, respectively (Table 4). Furthermore, no surviving bacteria were found even by a treatment with the high concentration of SAEW at both open and closed conditions.

**TABLE 4:** Bactericidal activity of slightly acidic electrolyzed water (SAEW) before and after 6 months of storage.

Samples	EC		SE		LM		SA		BC	
	Surviving population (log10 CFU/mL)									
	0 month	6 months	0 month	6 months	0 month	6 months	0 month	6 months	0 month	6 months
High-open	ND	ND	ND	ND	ND	ND	ND	ND	ND	ND
High-close	ND	ND	ND	ND	ND	ND	ND	ND	ND	ND
Low-open	ND	ND	ND	ND	ND	ND	ND	ND	ND	3.66 ± 0.16^a^
Low-close	ND	ND	ND	ND	ND	ND	ND	ND	ND	2.79 ± 0.06^b^

*Bars labeled with different letters in the same reduction group show a significant difference (p < 0.05).*

## Discussion

The growing population with its accompanying industrialization and economic development has increased the pressure upon both water resources and our land (Jiang et al., 2018). Industrial wastewater, pesticides, herbicides, and chemical fertilizers not properly contained have entered the soil, infiltrated some aquifers, and reduced the groundwater quality (Chung et al., 2015). Locally, this may change the water hardness and may affect the acceptability of drinking water.

Water samples were collected from 16 representative sites based on river reservoir, of which 14 samples were tap water, and 2 samples were from groundwater. The four rivers (Geum River, Nakdong River, Han River, and Yeongsan River) flow through an area where human activities thrive and, accordingly, are an important water source for human activities (Pandey et al., 2019). The collected sample showed different water hardness, because the hardness of water in those 16 sites exhibited different water hardness (Hori et al., 2021). Water hardness in the Gangwon-do, located on the north of South Korea, was lower to that in the Gyeongnam region. As Gangwon-do is a snowfall region, the raw water contains snowmelt water, which therefore showed low hardness (Lee et al., 2021). Nakdong River is the largest river in South Korea (length: 389 km), and its downstream area passes through Gyeongsang provinces and Busan city (Kim et al., 2021). The downstream region of the Nakdong River is highly polluted because of the accumulation of contaminants in the upstream (Venkatramanan et al., 2014). In addition, urbanization and human activities increase concentration of major ions such as Ca and Mg in the downstream areas (Cerqueira et al., 2020). For these reasons, the hardness of tap water in the Gyeongsang provinces and Busan city is relatively higher, compared with that in other places.

The EW generated from hardness less than 50 mg/L could not produce SAEW with accepted pH range from 5.0 to 6.5, whereas it was possible to produce proper SAEW from those water with hardness from 50 to 80 mg/L. All generator settings and other conditions were the same for producing SAEW except for water source. In this study, the main concern in SAEW production was the effect of hardness of starting water. High hardness means high content of CaCO_3_/MgCO_3_ in water (Park et al., 2018). The neutralization reaction (CaCO_3_ + 2HCl → CaCl_2_ + CO_2_ + H_2_O/MgCO_3_ + 2HCl → MaCl_2_ + CO_2_ + H_2_O) in number 4 bottle may be easier to occur in the high-hardness water samples. Therefore, the pH of EW would be increased. Both Forghani et al. (2015) and Kim et al. (2019) reported that higher water hardness led to higher pH of EW, which was consistent with our results. These results indicate that different water hardness should be considered while planning a sanitization approach for acquiring EW generators or food plant/facility.

Because of the different hardness of water source, some waters would have less potential to produce appropriate SAEW. Therefore, it is necessary to optimize the generation conditions by changing electrolyte concentrations or amperage or in addition to salts. The combination of 2% HCl and 2 M NaCl at 7 A showed proper pH and a higher germicidal effect of SAEW. The increase in NaCl concentration might augment electrolyte concentration, electrical current, or conductivity in the electrolytic solutions (Zhang et al., 2020). Moreover, there are chloride ions and hydroxyl ions to obtain electrons at the anode, for which chloride ions have strong ability to obtain electrons and are reduced to chlorine chloride ions. Meanwhile, there are hydrogen ions and sodium ions at the cathode, and hydrogen ions have a strong ability to lose electrons and oxidize into hydrogen (Rahman et al., 2016). In total, the following final reaction in the generation chamber at the presence of NaCl and HCl (2NaCl + 2H_2_O → 2NaOH + H_2_ + Cl_2_/2HCl → H_2_ + Cl_2_) can happen. Therefore, the increase in NaCl concentration might contribute to increasing pH value, which is important to produce of SAEW with acceptable the ACC and pH.

Electrolyzed water showed antimicrobial activity against a wide range of microorganisms and eliminate the most common forms of bacteria, fungi, viruses, and spores in food, food-processing surface, and non-food-contact surface in a relatively short time (Phuvasate and Su, 2010; Zheng et al., 2013). In our study, SAEW with an ACC of 27–41 mg/L for 1 min was sufficient to completely inactivate non–spore-forming foodborne pathogens (*E. coli*, *Salmonella* enteritidis, *L. monocytogenes*, and *S. aureus*). In addition, the SAEW against endospore-forming bacteria was also investigated. Within 1-min SAEW treatment, the extents of reduction were 3.62–3.93 log CFU/mL for *C. perfringens* spores and 8.23 log CFU/mL for *B. cereus* spores. These results were almost in agreement with recently reported study (Li et al., 2017; Liao et al., 2017; Luo et al., 2017). There is a significant difference in bactericidal effect between spore and non–spore formers by using SAEW. Kim et al. (2019) reported that SAEW (pH: 6, ACC: 20) can efficiently inactivate *E. coli O157:H7*, *S. aureus*, *Salmonella* enteritidis, and *B. cereus* with 10-min treatments. Al-Qadiri et al. (2019) also reported that the extent of reduction was 1.53 log CFU/mL for *B. cereus* and 1.8 log CFU/mL for *C. perfringens* after 1 min of treatment at 60 ppm, respectively. The main reason might be that most chemicals are limited to penetrate into the cytoplasm of spore-forming bacteria, which may provide an initial barrier for the penetration of active ingredients (disruption of cell membrane and denaturation of other organic macromolecules) (Kim et al., 2000).

Moreover, the application of SAEW is limited by some factors including storage condition. SAEW stored under closed conditions is more stable, whereas chlorine loss is found to be greater under open conditions (Ngnitcho et al., 2017). Our results were consistent with the reports of Rahman et al. (2012), showing that SAEW was more conducive to storage in a closed, dark container (Rahman et al., 2012). Cui et al. (2009) reported that the ACC of AEW completely decreased after 6 days of storage at open-dark or open-light condition. In addition, the pH of SAEW remained stable under the closed condition. As the solution is electrolytes, if kept open, the CO_2_ from the air might have consistently reacted with these electrolytes until equilibrium is reached; thus, the pH will rise. Therefore, SAEW should be stored in closed containers at higher ACC to maximize its effectiveness.

## Conclusion

Based on the study, water hardness is a key factor that should be taken into account whenever designing a procedure to produce SAEW. Overall, tap water having water hardness ranging from 50 to 80 ppm can produce proper SAEW. Low water hardness (<50 ppm) can be reinforced by adding the combination of HCl with NaCl. The electrolyte combination of 2% HCl and 2 M NaCl at 7A revealed proper pH and a higher germicidal effect of SAEW. High water hardness (>80 ppm) can be used based on the addition of 5% HCl. Comparison of basic properties and bactericidal efficiency of SAEW under two different conditions (open and close) before and after 6-month storage was also investigated. It indicated that the properties and antimicrobial effect of SAEW were stable at 40 ppm during storage. Further studies on the germicidal mechanisms *in vitro* and *in vivo* will be necessary.

## Data Availability Statement

The raw data supporting the conclusions of this article will be made available by the authors, without undue reservation.

## Author Contributions

PY: investigation, data curation, formal analysis, and writing—original draft. RC: writing—review and editing. KJ, H-YJ, NW, MW: investigation. DO: project administration and supervision. All authors contributed to the article and approved the submitted version.

## Conflict of Interest

H-YJ, NW, and MW were employed by Seoulin Bioscience Company. The remaining authors declare that the research was conducted in the absence of any commercial or financial relationships that could be construed as a potential conflict of interest.

## Publisher’s Note

All claims expressed in this article are solely those of the authors and do not necessarily represent those of their affiliated organizations, or those of the publisher, the editors and the reviewers. Any product that may be evaluated in this article, or claim that may be made by its manufacturer, is not guaranteed or endorsed by the publisher.

## Abbreviations

SAEW: slightly acidic electrolyzed water
ACC: available chlorine concentration
HCl: hydrochloric acid
NaCl: sodium chloride
PI: propidium iodide
SYT: Syto-9.
