# Supplementary material for: Optimization and Effect of Water Hardness for the Production of Slightly Acidic Electrolyzed Water on Sanitization Efficacy
Source: Front Microbiol. 2022 Mar 2;13:816671. doi: 10.3389/fmicb.2022.816671 (PMC8924475; doi:10.3389/fmicb.2022.816671)
Supplement: Supplementary file 1 [file Data_Sheet_1.docx]

Supplementary Material

#
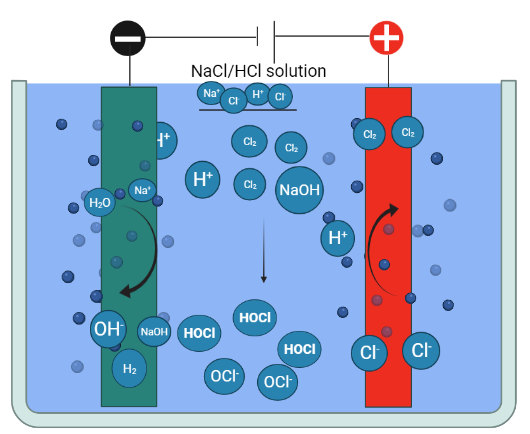


**Supplementary Figure 1.** The mechanism of SAEW generation using an electrolytic cell without diaphragm. The pH of SAEW is 5.0-6.5. There are the following reaction during the reaction: 2HCl→ H_2_ + Cl_2_, Cl_2_ + H_2_O → HOCl + HCl, 2NaCl + 2H_2_O → 2NaOH + H_2_ + Cl_2_, Cl_2_ + 2NaOH → HOCl + NaCl + NaOH, HCl + NaOH → NaCl + H_2_O.

.


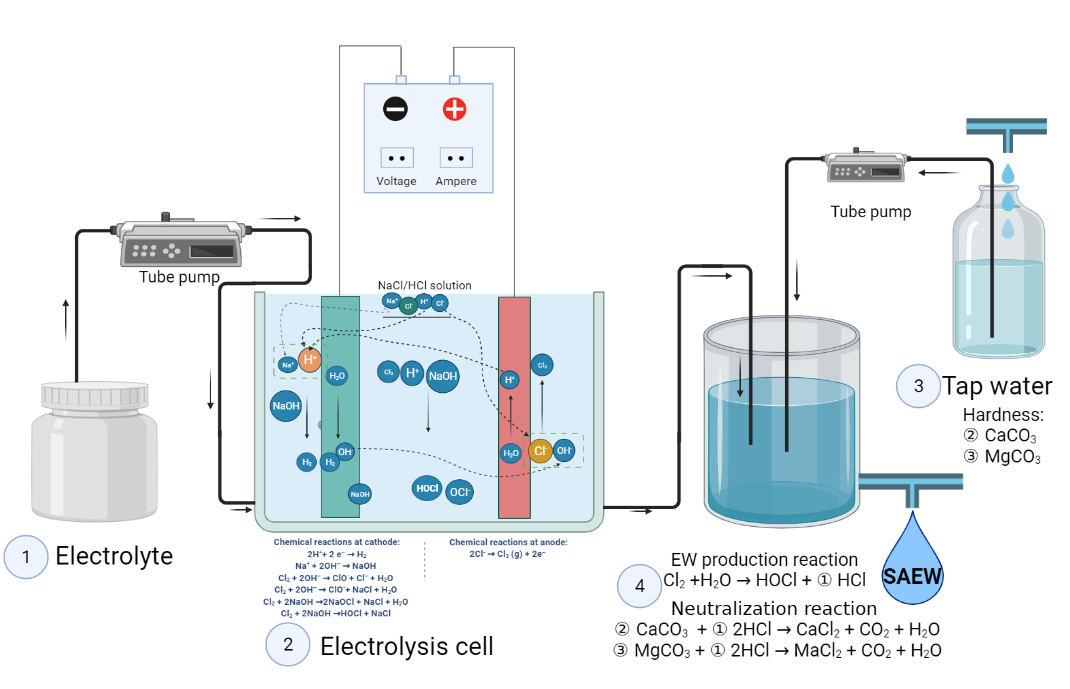


**Supplementary Figure 2.** Schematic illustration of the slightly acidic electrolyzed water generator system used to produce SAEW in the study. Number 1: electrolyte, Number 2: electrolytic cell, Number 3: water tank (tap water from different places), Number 4: SAEW produced. Processing description: electrolyte solution flows into the electrolysis cell by turning up the tube pump switch power supply, the reaction is being happened (Chemical reactions at cathode: 2H++ 2 e- → H_2_, Na^+^ + 2OH^-^ → NaOH, Cl_2_ + 2OH- → ClO^-^+ Cl^-^ + H_2_O, Cl_2_ + 2OH- → ClO^-^+ NaCl + H_2_O, Cl_2_ + 2NaOH →2NaOCl + NaCl + H_2_O, Cl_2_ + 2NaOH →HOCl + NaCl; Chemical reactions at anode:2Cl- → Cl_2_ (g) + 2e^-^), then the solution flows into the number 4 happening the SAEW producing reaction(Cl_2_ +H_2_O → HOCl + HCl). Finally, SAEW is generated by EW generator system.


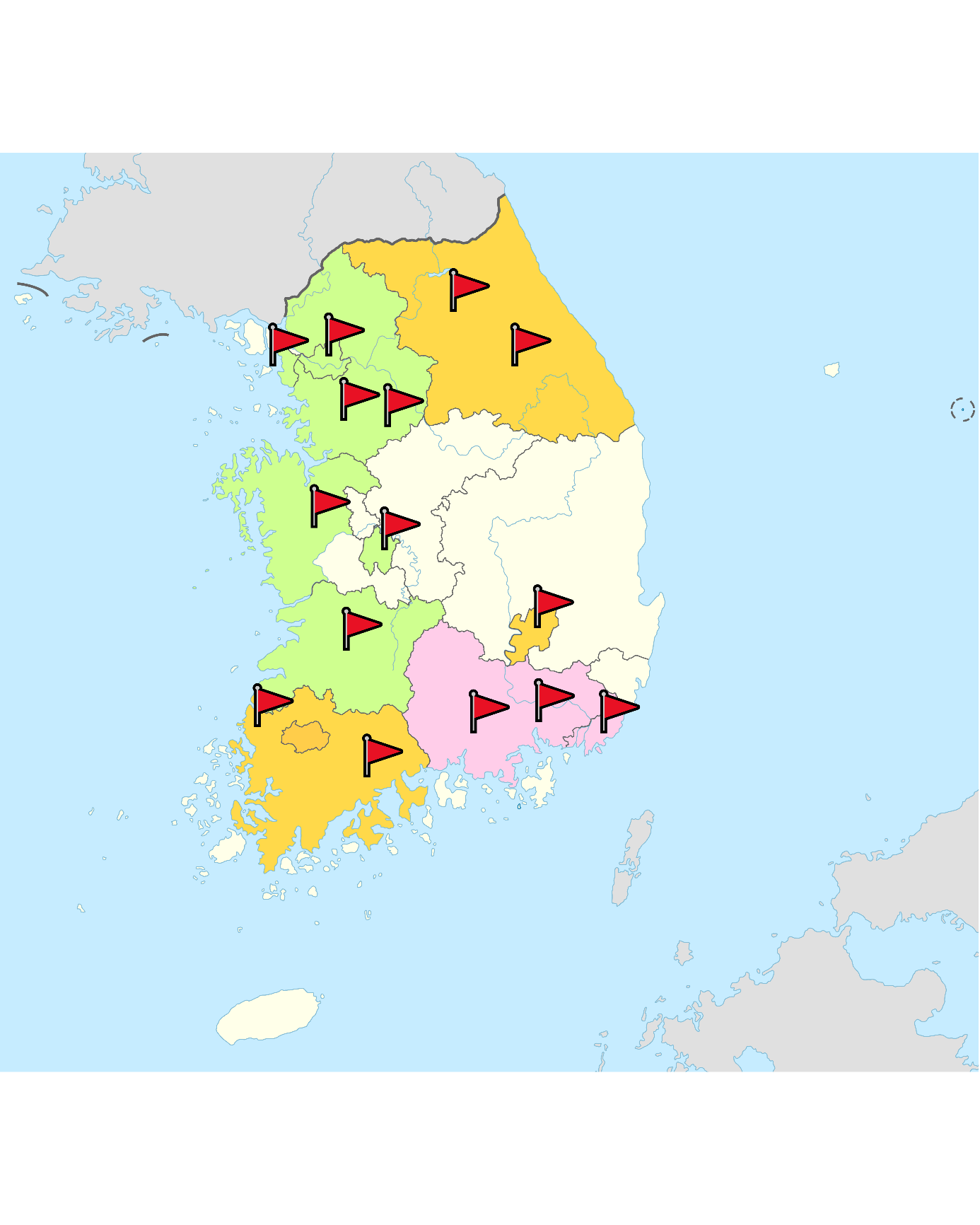


**Supplementary Figure 3.** Location map of collected water samples in the South Korea. The yellow color of region: low water hardness (21-41 ppm); the green color of region: middle water hardness (50-80 ppm); the pink color of region: high water hardness (>80 ppm).


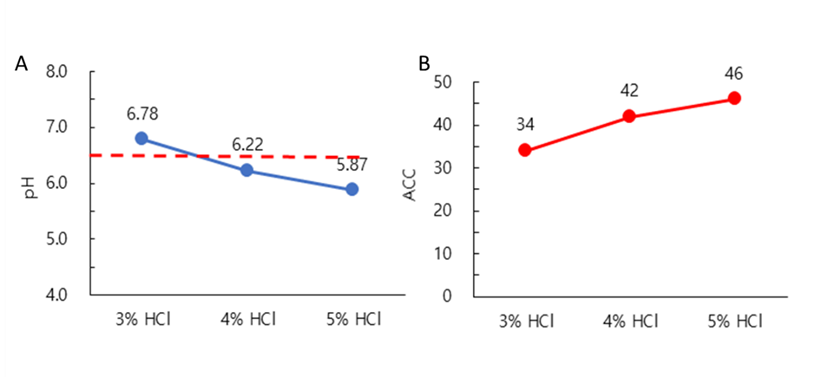


**Supplementary Figure 4.** Optimization of SAEW production system by increasing the concentration of HCl using high hardness water (212 ppm). A: Relation of addition of HCl to pH of producing EW; B: Relation of addition of HCl to ACC.
